# Association of Healthy Eating Index-2015 Total and Component Scores with Measures of Inflammation and Immune Activation in Healthy Adults

**DOI:** 10.1016/j.tjnut.2025.01.005

**Published:** 2025-01-07

**Authors:** Charles B Stephensen, Xiaowen Jiang, Barbara Gale, Janet M Peerson

**Affiliations:** 1 USDA Western Human Nutrition Research Center, Davis, CA, United States; 2 Nutrition Department, University of California, Davis, CA, United States

**Keywords:** dietary pattern, immunity, inflammation, healthy eating index (HEI), visceral fat mass

## Abstract

**Background:**

The Healthy Eating Index (HEI)-2015 measures diet quality and is associated with a lower risk of death from chronic disease. Dietary components may affect health via multiple mechanisms, including decreasing inflammation and affecting immune activation.

**Objective:**

We hypothesized that the overall HEI-2015 score, or individual component scores, would be associated with altered inflammation and immune activation in healthy adults.

**Methods:**

The association of HEI-2015 scores with 88 inflammation and immune activation markers was examined in 346 adults without diagnosed disease using general linear models to adjust for covariates, including visceral fat mass index (VFMI).

**Results:**

The overall HEI-2015 score was inversely associated with plasma C-reactive protein (CRP) and leukocyte concentrations, which are markers of inflammation, but these associations lost statistical significance with adjustment for VFMI. However, even with VFMI adjustment, the total vegetable score was inversely associated with total lymphocyte concentration (β = -0.157 ± 0.052, *P* = 0.019) and with monocyte and neutrophil activation (e.g., classic monocyte CD11b β = −0.153 ± 0.055, *P* = 0.030; neutrophil CD11b β = −0.122 ± 0.051, *P* = 0.049). The refined grain score was inversely associated with percent NK-T cells (β =−0.171 ± 0.058, *P* = 0.037), IL-10 production by T cells (β = −0.204 ± 0.057, *P* = 0.0039), and positively associated with plasma soluble CD14 (β = 0.220 ± 0.059, *P* = 0.0041). The total dairy score was positively associated with production of multiple cytokines by lipopolysaccharide-stimulated peripheral blood mononuclear cells [e.g., interleukin (IL)-1β β = 0.182 ± 0.054, *P* = 0.0066].

**Conclusions:**

Adjustment for VFMI decreased the association of HEI-2015 with inflammation, consistent with the known role of adiposity in mediating effects of poor diet on inflammation. This study also identified component scores associated with various aspects of immune activation that bear further study to clarify possible health benefits.

This trial was registered at clinicaltrials.gov as NCT02367287**.**

## Introduction

Healthy dietary patterns can lead to better health outcomes. Evidence documenting this relationship forms the basis of the Dietary Guidelines for Americans [1]. The Healthy Eating Index (HEI) was first developed in 1995 [2] as an index of overall diet quality to use in monitoring actual dietary patterns relative to the Dietary Guidelines for Americans. The Guidelines and the HEI scoring system were jointly updated in 2005 [3], 2010 [4], and 2015 [5]. In 2020, the Dietary Guidelines were updated, but the HEI-2015 scoring system was not changed, although it was renamed HEI-2020 to correspond to the Dietary Guidelines for Americans, 2020–2025 [6]. Many studies have demonstrated the positive association of diet quality with health outcomes. Evaluation of diet quality using HEI-2015 in the NIH-AARP Diet and Health Study cohort study found a 13%–23% lower risk of mortality from all causes, cancer, and cardiovascular disease in the highest compared with the lowest HEI-2015 quintiles [7]. A similar analysis from the Nurses Health Studies and the Health Professionals Follow-up Study found that a 25-point higher HEI-2015 score (of 100 total points) was associated with a 20% lower risk of incident cardiovascular disease, with other dietary quality scores showing similar associations [8]. A recent literature review also supports an association of better diet quality with lower cardiovascular disease risk [9]. Higher HEI-2015 scores have also been associated with lower risk of metabolic dysfunction-associated fatty liver [10] as well as lower risks of obesity, hyperglycemia, hypercholesterolemia, and high blood pressure in the US NHANES [11].

Healthy diets may decrease the risk of chronic disease by a variety of mechanisms. One mechanism may be reduction of inflammation or immune activation. Several community-based, observational studies of generally healthy adults have examined the association between HEI and markers of inflammation [[12], [13], [14], [15], [16], [17], [18]]. Five of these studies were included in a systematic literature review that also reviewed the use of other dietary indexes [19]. These studies primarily used serum or plasma markers of inflammation including the acute phase proteins C-reactive protein (CRP) and serum amyloid A (SAA), cytokines including IL-6 and TNF-a, adhesion molecules including intercellular adhesion molecule (ICAM)-1, vascular cell adhesion molecule (VCAM)-1, E-selectin, the coagulation factor fibrinogen, and complete blood count (CBC) data providing blood leukocyte concentrations and percentages. Results differed across studies, but some studies showed that higher HEI scores were associated with lower inflammation and leukocyte concentrations. To clarify associations of diet with immune function, these studies often adjusted for demographic and other personal characteristics known to be associated with immune function, such as age, sex, BMI (as a proxy for adiposity), and physical activity. The goal of the present study was to examine the association of the HEI-2015 total and component scores with a broad set of 88 markers of inflammation and immune activation in 346 participants of the Nutritional Phenotyping Study [20] conducted at the USDA Western Human Nutrition Research Center (WHNRC). This set of 88 markers, as previously described [21], includes measures of both innate and adaptive immunity, as well as plasma markers of inflammation. Their use thus allows a broader characterization of diet with immune activation than has previously been reported using the HEI.

## Methods

### Study design

Healthy adults were recruited into the cross-sectional Nutritional Phenotyping Study (clinicaltrials.gov NCT02367287) conducted at the USDA WHNRC in Davis, California, from May 2015 through July 2019. The study design [20] and Consolidated Standards of Reporting Trials diagram reporting participant recruitment and allocation [22] have been published. The sample size target was 396 adults (22 individuals in each of 18 recruitment bins), and 393 were enrolled. The sample size calculation was designed to detect associations between dietary variables and specific outcomes (e.g., immune variables), explaining ∼3% of the variation in specific associations. Recruitment was stratified across time into 18 bins using 3 age categories (18–33, 34–49, and 50–65 y) and 3 BMI categories (18.5–24.9, 25–29.9, and 30–45.0 kg/m^2^) within each category, for men and women separately. The first visit required approximately half a day and included informed consent, measurement of weight and height, dual-energy X-ray absorptiometry (DXA) to assess body composition, instructions for completing ASA24 dietary recalls in the ensuing interval before visit 2, and other study instructions. Visit 2 was preceded by a standard, study-provided meal the evening before the visit, followed by a 12 h overnight fast. Fasting blood was drawn at the beginning of visit 2, which lasted ∼9 h. Of the 393 participants enrolled at visit 1, fasting blood was available from 362 participants, and 358 participants substantially completed all day 2 activities.

### 24-h dietary recalls

Of the 393 participants enrolled at visit, 1378 completed at least 2 of the 3 desired ASA24 at-home recalls. Manual data cleaning and comparison of total energy intake were compared with total energy expenditure measured via activity monitoring to assess the quality of the dietary intake data, as described [23]. The 2015 HEI was determined for the 378 participants with 2 at-home recalls as previously reported [22]. This analysis used the HEI total score as well as the 13 component scores that are summed to create the total score (total point range 0–100 for the total score). HEI scores were calculated using programs provided by the National Cancer Institute (Institute, N. C. *Healthy Eating Index SAS Code*. https://epi.grants.cancer.gov/hei/sas-code.html [accessed 2018-06-16]).

### DXA determination of visceral adipose tissue

Magnetic resonance imaging (MRI) provides 3-dimensional data to directly estimate visceral adipose tissue (VAT) volume, but recent studies have shown that use of 2-dimensional DXA data can be used to estimate VAT volumes that correlates highly with MRI VAT volume results with *R* values >0.90 [24,25]. DXA scans were performed on study participants at visit 1 using a Discovery W (S/N 84994) model Hologic DXA whole-body scanner and Version 13.6.0.7 software. Scans were available from 390 study participants. After completion of the study, these scans were used to estimate VAT volume, area, and mass by a single, experienced DXA operator selecting appropriate anatomical landmarks. The estimated VAT weights were used to create a VAT mass index (VFMI) by dividing VAT mass in grams by height in meters squared (g/m^2^). Within sex, this index was not associated with height. Sex-specific VFMI tertiles were determined for these 390 study participants (Supplemental Table 1) for use in categorical data analysis.

### Immune variables

A novel set of 88 immune variables was used to characterize the “immune phenotype” of study volunteers. These variables have been previously described [21]. In brief, immune assessment used fasting blood drawn at visit 2 from 362 participants. These variables included 6 sets of markers: *1*) 19 plasma markers consisting of acute phase proteins, soluble adhesion molecules, cytokines, chemokines, matrix metalloproteinases, neopterin, myeloperoxidase, and soluble CD14; *2*) 15 variables from a complete blood counts (CBCs) with differential assessment of leukocyte types; *3*) 11 markers of innate immune cell activation assessed by flow cytometric analysis of whole blood; *4*) 24 markers of CD4 and CD8 T-cell phenotypes and activation level assessed by flow cytometric analysis of isolated peripheral blood mononuclear cells (PBMCs); *5*) 11 markers of CD4 T-cell, B cell, NK, and NK T-cell phenotypes; and *6*) 8 markers consisting of the concentrations of 4 T-cell cytokines produced by PBMC cultures stimulated in a T-cell-specific manner (using anti-CD3 and anti-CD28 antibody stimulation) and 4 cytokines produced by PBMC cultures stimulated with bacterial LPS.

### Cytomegalovirus infection history

Cytomegalovirus (CMV) seropositivity was assessed by determining the presence of CMV-specific IgG antibody in plasma (Roche Diagnostics), as previously described [21]. Results were characterized as positive (*n* = 179), borderline (*n* = 1), or negative (*n* = 181). The one borderline sample was considered negative for the present analysis (total *n* = 361).

### Physical activity assessment

Participants were instructed to wear an Actical (Philips Respironics) activity monitor attached to a belt positioned over their left hip for ≥7 d. Of the 347 study volunteers with Actical data used in this report, 8 volunteers had 3 or 4 d of data, 326 had 5–9 d of data, and 13 had 10–23 d of data from wearing the Actical. The Actical converts movement (activity counts) into energy units (calories) and categorizes each time interval (set to 1 min) into 1 of 4 activity categories: sedentary, light, moderate, or vigorous activity corresponding to 0, 0.001–0.041, 0.042–0.099, or ≥0.1 kcal/kg body weight per minute. The number of minutes in each category was determined for a full day of 1440 min. Incomplete days (i.e., <12 h of time recorded per day) were not used for statistical analysis. Personal sleep logs were also used to identify periods of sleep that were considered sedentary activity. The mean percent of time for each of the 4 activity periods was used to determine the mean daily minutes in each period for the full period of activity monitoring for each participant.

### Statistical analysis

A general linear model procedure (PROC MIXED or PROC GLIMMIX) was used in SAS statistical analysis software (version 9.4, SAS Institute). The HEI total and individual component scores were each used as independent variables to predict the value for each of the 88 dependent variables. The model used covariates including sex, age category (18–33, 34–49, and 50–65 y), VFMI category (sex-specific tertiles), month of year (categorical), CMV status (positive/negative), and fitness (3 continuous terms for light, moderate and vigorous activity). Interaction terms with HEI (a continuous variable) were used for age, sex, and VFMI categories but not for CMV, fitness, or month. Immune variables were transformed, if necessary, to normalize their distribution. These transformations and sample sizes for each regression model are shown in Supplemental Table 2. *P* values for significance of β-coefficients were determined independently for each model (i.e., for the HEI total score plus 13 component scores for each immune variable) without and with adjustment for multiple comparisons using the Benjamini-Hochberg method and sample sizes within each of the following 6 immune variable groups, as indicated here: *1*) 19 plasma variables; *2*) 15 CBC variables; *3*) 11 variables of innate immune cell activation; *4*) 24 variables of CD4 and CD8 T-cell phenotypes; *5*) 11 variables of CD4 T-cell, B cell, NK and NK T-cell phenotypes; and *6*) 8 variables for cytokine production by PBMC cultures. We report on 2 variations of this general linear model in this manuscript. Model 1 was as described above but did not include VFMI as a covariate. Model 2 was identical to model 1 but included VFMI as a covariate. Figures show statistical significance with and without adjustment for multiple comparisons, but only significant associations with adjustment for multiple comparisons are mentioned in the Results and Discussion sections. Standardized β-coefficients, SEs, and unadjusted and adjusted *P* values for each model are shown in Supplemental Table 3. Multiple analysis of variance was used to compare minutes of activity within each activity category (sedentary, light, moderate, and vigorous) across the sex, age, and VFMI categories without adjustment for covariates.

Sample sizes for each analysis included ≤346 participants. The number of volunteers in the sex, age, CMV status, and VFMI categories are shown in Table 1, along with the race/ethnicity of the study participants.

**TABLE 1:** Numbers of participants (with immune data available) grouped by sex, cytomegalovirus antibody status (CMV Status), and race/ethnicity within the 3 study recruitment categories (sex, age, BMI) and sex-specific visceral fat mass index tertiles.

Categories	All	Sex		Age Category (y)			BMI Category (kg/m^2^)			VFMI (g/m^2^) Tertiles		
		Male	Female	18–33	34–49	50–66	18–25	25–30	30–44	1	2	3
	*N* = 346	*N* = 164	*N* = 182	*N* = 117	*N* = 117	*N* = 112	*N* = 136	*N* = 124	*N* = 86	*N* = 118	*N* = 117	*N* = 111
Sex												
Female, *n* (%)	182 (52.6)		182 (100)	62 (53.0)	58 (49.6)	62 (55.4)	70 (51.5)	62 (50.0)	50 (58.1)	65 (55.1)	61 (52.1)	56 (50.5)
Male, *n* (%)	164 (47.4)	164 (100)		55 (47.0)	59 (50.4)	50 (44.6)	66 (48.5)	62 (50.0)	36 (41.9)	53 (44.9)	56 (47.9)	55 (49.5)
CMV Status												
Negative, *n* (%)	170 (49.1)	75 (45.7)	95 (52.2)	54 (46.2)	63 (53.8)	53 (47.3)	70 (51.5)	57 (46.0)	43 (50.0)	57 (48.3)	57 (48.7)	56 (50.5)
Positive, *n* (%)	176 (50.9)	89 (54.3)	87 (47.8)	63 (53.8)	54 (46.2)	59 (52.7)	66 (48.5)	67 (54.0)	43 (50.0)	61 (51.7)	60 (51.3)	55 (49.5)
Race/Ethnicity												
White, *n* (%)	213 (61.6)	97 (59.1)	116 (63.7)	48 (41.0)	85 (72.6)	80 (71.4)	86 (63.2)	72 (58.1)	55 (64.0)	73 (61.9)	67 (57.3)	73 (65.8)
Hispanic, *n* (%)	46 (13.3)	21 (12.8)	25 (13.7)	22 (18.8)	12 (10.3)	12 (10.7)	16 (11.8)	15 (12.1)	15 (17.4)	15 (12.7)	12 (10.3)	19 (17.1)
Black, *n* (%)	14 (4.0)	7 (4.3)	7 (3.8)	7 (6.0)	3 (2.6)	4 (3.6)	1 (0.7)	7 (5.6)	6 (7.0)	5 (4.2)	5 (4.3)	4 (3.6)
Asian, *n* (%)	46 (13.3)	25 (15.2)	21 (11.5)	29 (24.8)	9 (7.7)	8 (7.1)	24 (17.6)	18 (14.5)	4 (4.7)	17 (14.4)	21 (17.9)	8 (7.2)
American Indian, Pacific Islander, *n* (%)	2 (0.6)	1 (0.6)	1 (0.5)	0 (0)	1 (0.9)	1 (0.9)	0 (0)	1 (0.8)	1 (1.2)	0 (0)	0 (0)	2 (1.8)
Other, Multiple Groups, Declined, *n* (%)	25 (7.2)	13 (7.9)	12 (6.6)	11 (9.4)	7 (6.0)	7 (6.3)	9 (6.6)	11 (8.9)	5 (5.8)	8 (6.8)	12 (10.3)	5 (4.5)

## Results

### Participant characteristics

The design of the Nutritional Phenotyping Study balanced recruitment of healthy adults by sex, age category, and BMI category as a proxy for adiposity. This balanced design resulted in the mean BMI being the same for men and women as well as for the 3 age groups, whereas mean BMI, of course, differed by BMI category (Supplemental Figure 1A). In the present analysis, we chose to use VFMI as an adiposity index. As expected, mean VFMI was higher in each BMI category, increasing in a stepwise fashion (Supplemental Figure 1B). Mean VFMI, similar to BMI, did not differ by sex but, unlike BMI, was higher in the 2 older age groups than in the youngest age group.

Significant differences in activity were seen between men and women and among the age and VFMI categories (Supplemental Figure 2). Women had lower moderate activity than men, the 2 older age categories had lower sedentary but higher light activity than the youngest category, whereas VFMI tertile 3 had higher sedentary and lower moderate and vigorous minutes than tertiles 1 and 2.

Diet quality, as indicated by HEI scores, did not differ between men and women but did differ by age, with the oldest age group having a higher mean HEI score than the 2 younger age groups (Supplemental Figure 3), as has been previously reported [26]. HEI means differed among the 3 VFMI tertile groups, with HEI means being lower in the higher tertiles (Supplemental Figure 3). The component HEI scores generally showed the same patterns. For sex, only 3 scores differed, with total fruit and whole fruit scores being higher in women than men, but the saturated fat score was lower in women than men (Supplemental Figure 3). For age groups, the oldest group had higher component scores than the youngest group for total fruit, whole fruit, sodium, and refined grains, with the middle age group being in between (although the middle age group tended to be lower than the other 2 for whole grain and added sugars). For the VFMI tertiles groups, lower scores were seen in the highest compared with the lowest tertiles for total vegetables, greens and beans, total fruit, whole fruit, whole grain, seafood and plant protein, fatty acids, and saturated fats. Total dairy was the exception to this pattern, with the highest VFMI tertile having a higher score than the lowest tertile.

### Association of HEI scores with inflammation and immune activation

#### Plasma immune variables

Markers of inflammation, immune activity, and tissue damage were measured in plasma. Several statistically significant associations (after adjustment for multiple comparisons within this group of variables) were found between HEI scores and plasma immune markers using model 1. The strongest associations were found for CRP, where the total HEI score and scores for whole grains, fatty acids, and saturated fats were inversely associated with CRP concentration (Supplemental Figure 4). Significant inverse associations were also seen between the fatty acid and saturated fat scores and plasma MPO concentration. Two positive associations were seen between the refined grain score and sCD14 and between the saturated fat score and MMP-3. However, when model 2 was used to additionally adjust for VFMI, these associations were significantly attenuated, and only the positive association of refined grains with sCD14 (β = 0.220 ± 0.059, *P* = 0.0041) remained statistically significant with adjustment for multiple comparisons (Figure 1). The overall number of significant associations (with adjustment for multiple comparisons) decreased from 8 (6 inverse, 2 positive) using model 1 to 1 (positive) using model 2, representing an 88% decrease because of adjustment for VFMI.

**FIGURE 1:**
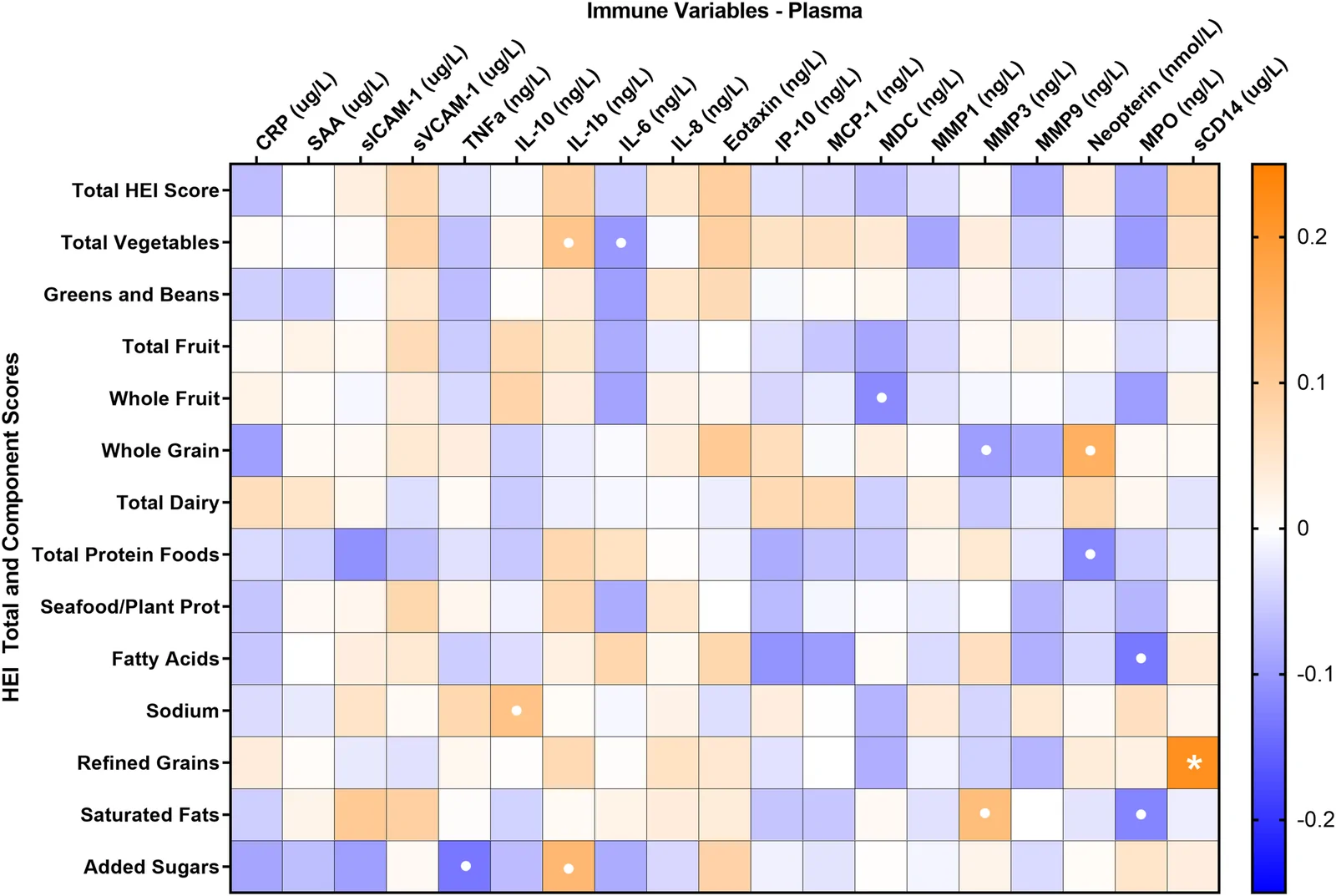
Heat map of β-coefficients from generalized linear regression models showing association of total and component Healthy Eating Index (HEI) scores with 19 plasma immune variables adjusting for age, sex, visceral fat mass index, season (using month of year), and cytomegalovirus immune status. Significant associations with and without adjustment for multiple comparisons are shown with an asterisk (∗) or dot (^•^), respectively. Sample sizes for each analysis are shown in Supplemental Table 2, and β-coefficients, SEs, and *P* values are shown in Supplemental Table 3.

### Blood leukocytes

Several statistically significant associations were seen for total leukocytes and 3 of the components, neutrophils, lymphocytes, and monocytes, using model 1 (Supplemental Figure 5). The total HEI score was inversely associated with total leukocytes, neutrophils, and lymphocytes; the total vegetable score was inversely associated with lymphocytes; the greens and beans score was inversely associated with total leukocytes, neutrophils, lymphocytes, and monocytes; and the saturated fats score was inversely associated with total leukocytes, neutrophils, and lymphocytes. The total vegetables score and greens and beans score were also positively associated with the platelet: lymphocyte ratio, presumably because of the inverse association of these scores with lymphocytes and the lack of a significant association with platelet concentration. The addition of VFMI to the model attenuated these associations (Figure 2), but significant, inverse associations remained between the total vegetable score (β = −0.157 ± 0.052, *P* = 0.019) and the greens and beans score (β = −0.173 ± 0.050, *P* = 0.010) and lymphocyte concentration. Similarly, the positive associations between these 2 scores and the platelet: lymphocyte ratio also remained significant (β = 0.183 ± 0.053, *P* = 0.010 and β = 0.153 ± 0.053, *P* = 0.035, respectively). The overall number of significant associations decreased from 13 (11 inverse, 2 positive) using model 1 to 4 using model 2 (2 inverse, 2 positive), representing a 69% decrease because of adjustment for VFMI.

**FIGURE 2:**
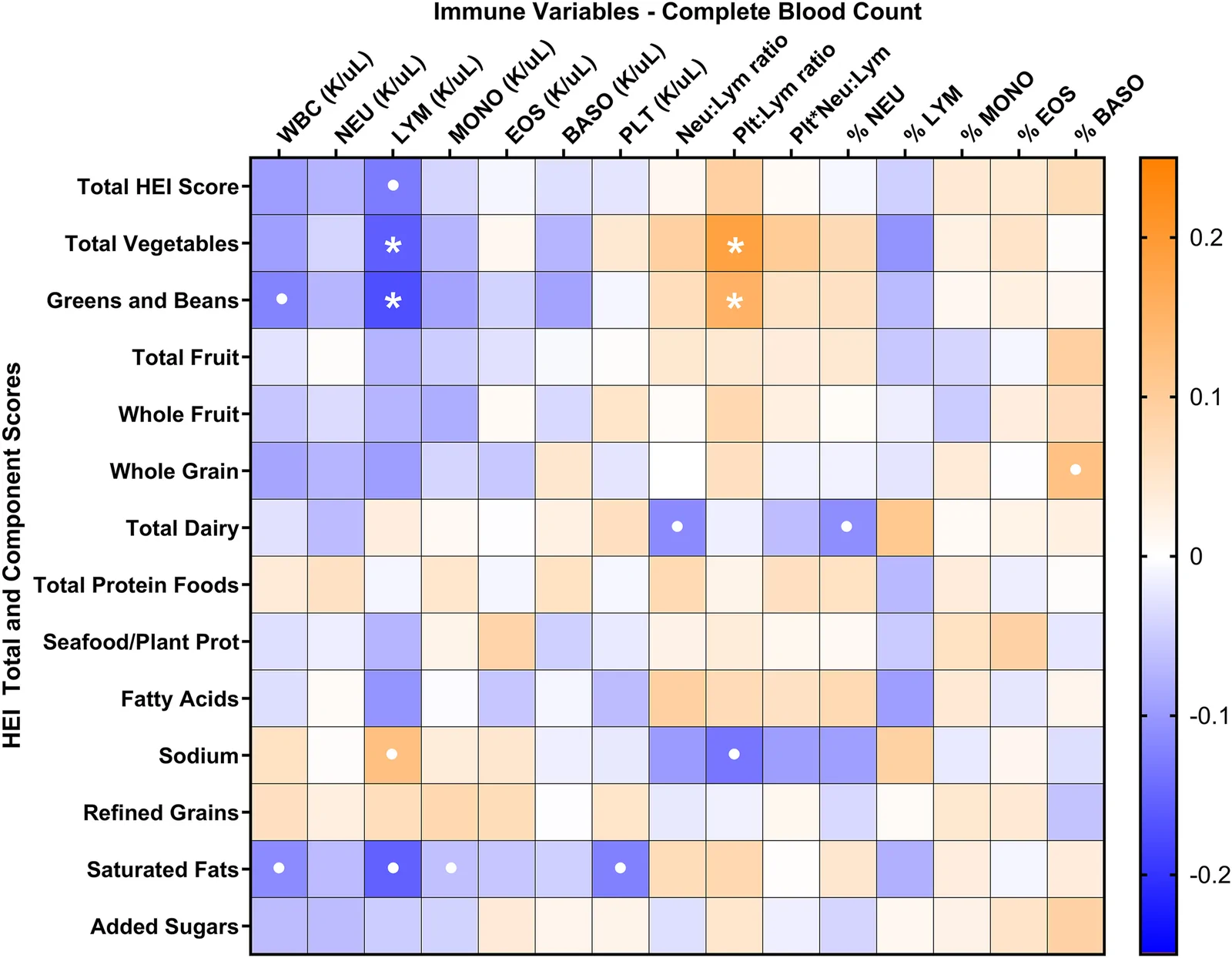
Heat map of standardized β-coefficients from general linear models showing association of total and component Healthy Eating Index (HEI) scores with 15 complete blood count immune variables adjusting for age, sex, visceral fat mass index, season (using month of year) and cytomegalovirus immune status. Significant associations with and without adjustment for multiple comparisons are shown with an asterisk (∗) or dot (^•^), respectively. Sample sizes for each analysis are shown in Supplemental Table 2, and β-coefficients, SEs, and *P* values are shown in Supplemental Table 3.

#### Innate leukocyte types and activation level

Flow cytometry was used to assess monocyte types and expression of activation markers on monocytes, neutrophils, and eosinophils in whole blood. The total vegetables score showed significant, inverse associations with the expression of CD11b on all 3 monocyte types (classic, intermediate, and alternative) using model 1 (Supplemental Figure 6). These associations remained significant with the addition of VFMI as a covariate (β = −0.153 ± 0.055, *P* = 0.030 for classic; β = −0.155 ± 0.054, *P* = 0.030 for intermediate; and β = −0.132 ± 0.051, *P* = 0.036 for alternative), and an additional significant, inverse association was seen with CD11b expression on neutrophils (β = −0.122 ± 0.051, *P* = 0.049) (Figure 3). A significant inverse association of the fatty acid score with percent intermediate monocytes that was seen with model 1 was attenuated and no longer statistically significant with model 2. The overall number of significant associations decreased from 4 in model 1 to 3 in model 2, representing a 25% decrease with adjustment for VFMI.

**FIGURE 3:**
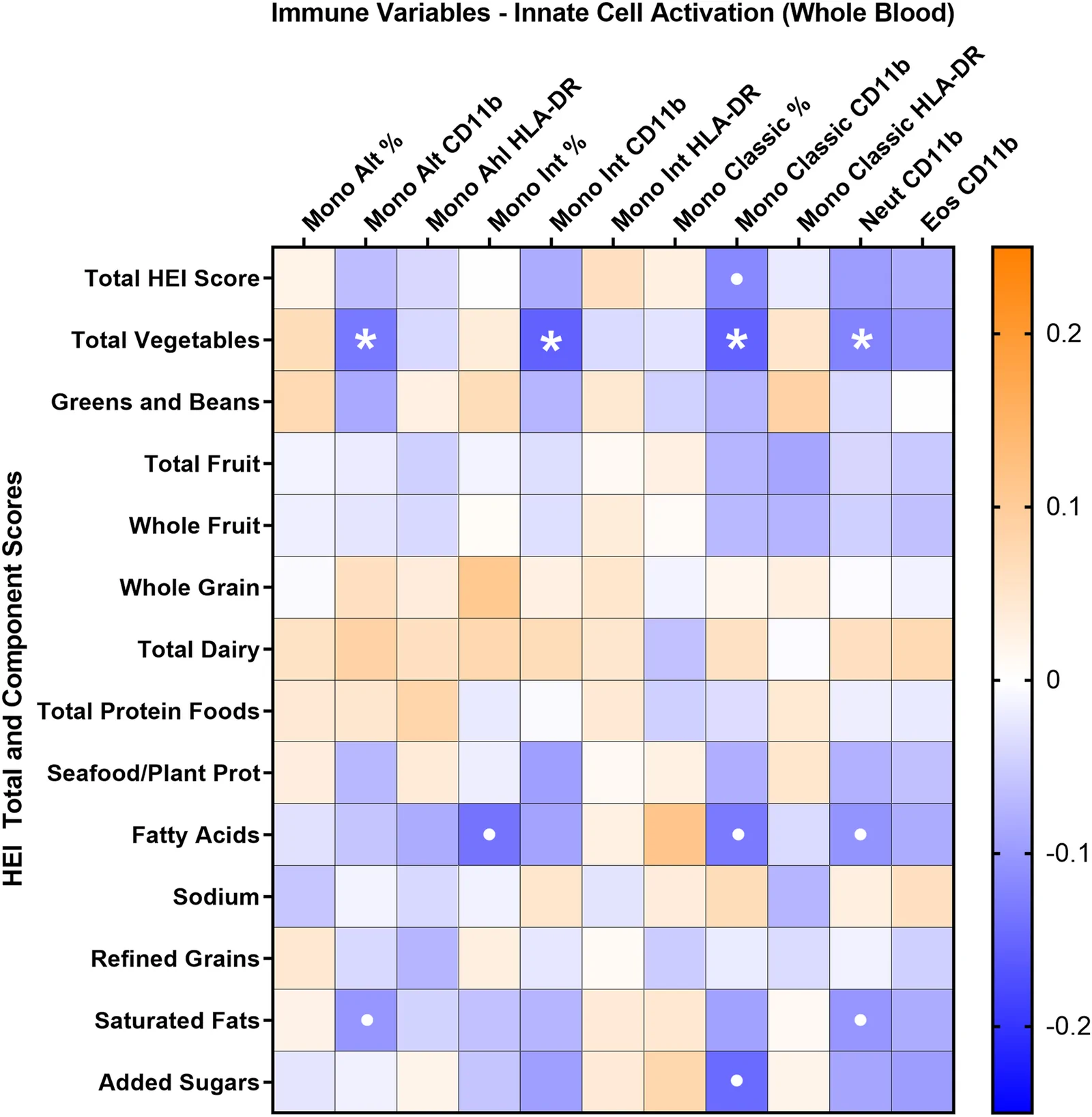
Heat map of standardized β-coefficients from general linear models showing association of total and component Healthy Eating Index (HEI) scores with 11 immune variables showing activation levels of monocyte types (Alternative, Intermediate, and Classical), neutrophils, and eosinophils adjusting for age, sex, visceral fat mass index, season (using month of year) and cytomegalovirus immune status. Significant associations with and without adjustment for multiple comparisons are shown with an asterisk (∗) or dot (^•^), respectively. Sample sizes for each analysis are shown in Supplemental Table 2, and β-coefficients, SEs, and *P* values are shown in Supplemental Table 3.

### T-cell percentages, subtypes, and activation levels

Flow cytometry was used to assess T-cell subsets and levels of activation using multiple markers on isolated PBMC. No statistically significant associations were found between total or component HEI scores using model 1 (Supplemental Figure 7) or using model 2, which included adjustment for VFMI (Figure 4). The overall number of significant associations thus did not differ because of adjustment for VFMI.

**FIGURE 4:**
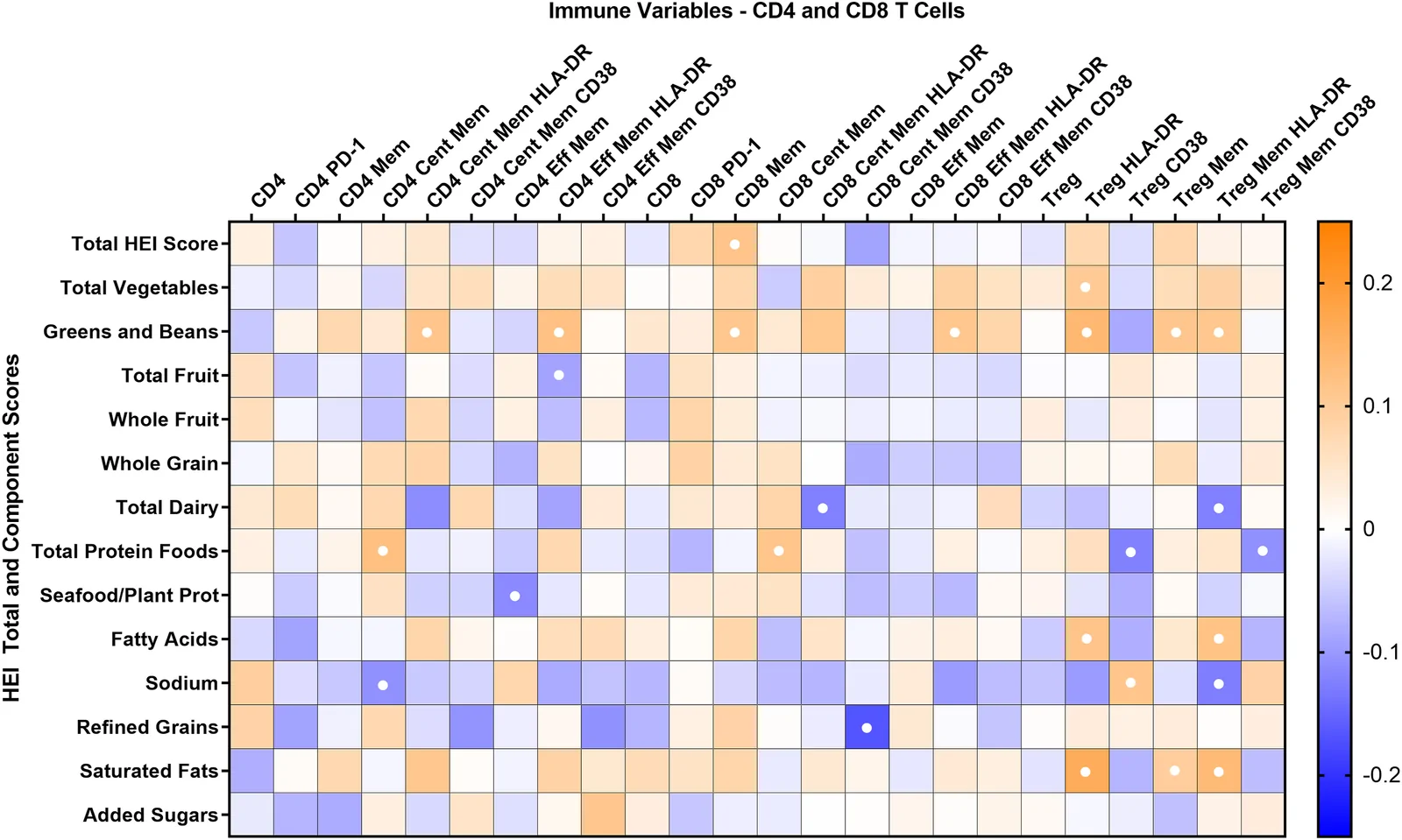
Heat map of standardized β-coefficients from general linear models showing association of total and component Healthy Eating Index (HEI) scores with 24 immune variables for CD4 and CD8 T-cell types (memory, central memory, effector memory) and subsets showing activation (HLA-DR and CD38) and exhaustion (PD1) marker expression adjusting for age, sex, visceral fat mass index, season (using month of year) and cytomegalovirus immune status. Significant associations with and without adjustment for multiple comparisons are shown with an asterisk (∗) or dot (^•^), respectively. Sample sizes for each analysis are shown in Supplemental Table 2, and β-coefficients, SEs, and *P* values are shown in Supplemental Table 3.

#### T-helper cells, B cells, and NK cells

Flow cytometry was used to identify these cell types isolated PBMC. Only 1 significant association was found, with the same association identified in both models. The refined grains score was inversely associated with NK T-cells in both model 1 (Supplemental Figure 8) and model 2 (β = −0.171 ± 0.058, *P* = 0.037) after adjustment for VFMI (Figure 5). The overall number of significant associations thus did not differ because of adjustment for VFMI.

**FIGURE 5:**
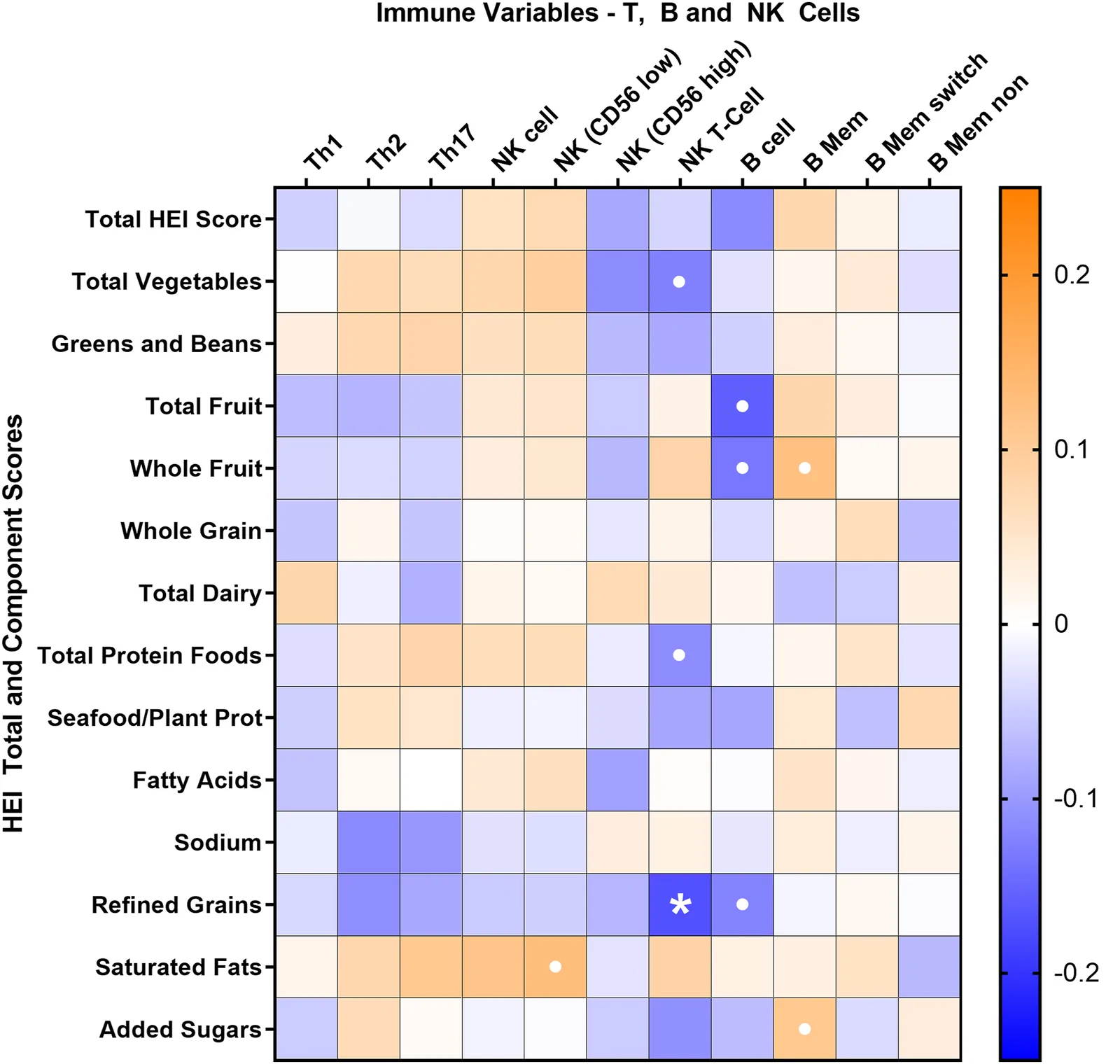
HEAT map of standardized β-coefficients from general linear models showing association of total and component Healthy Eating Index (HEI) scores with 11 immune variables for CD4 T-cells (TH1, Th2, TH17), NK cells and B cells adjusting for age, sex, visceral fat mass index, season (using month of year) and cytomegalovirus immune status. Significant associations with and without adjustment for multiple comparisons are shown with an asterisk (∗) or dot (^•^), respectively. Sample sizes for each analysis are shown in Supplemental Table 2, and β-coefficients, SEs, and *P* values are shown in Supplemental Table 3.

#### Production of cytokines by PBMC cultures

PBMCs were separately stimulated to produce cytokines as an indicator of responsiveness to prototypical stimuli for the innate and adaptive immune systems. For the innate system, PBMC with stimulated bacterial (*E. coli*) LPS stimulates toll-like receptor 4 (TLR4), a pattern recognition receptor of the innate immune system. For the adaptive system, PBMCs were stimulated with antibodies against 2 surface proteins, CD3 and CD28, which cause polyclonal stimulation of T-cells via the T-cell receptor. Regression analysis showed similar associations using models 1 and 2. For both models, significant inverse associations were seen between the whole grain score and T cell-stimulated IL-10 concentration (model 2 β = −0.204 ± 0.057, *P* = 0.0030) and between the fatty acids score and LPS-stimulated IL-1β concentration (model 2 β = −0.163 ± 0.056, *P* = 0.0031) (Supplemental Figure 9 and Figure 6). A significant, inverse association between the fatty acids score and LPS-stimulated IL-6 seen in model 1 was attenuated in model 2 and was no longer significant. Finally, significant positive associations were seen between the total dairy score and all 4 LPS-stimulated cytokines (TNF-α, IL-6, IL-1β, and IL-10) using both model 1 and model 2 (model 2 results for TNF-α, IL-6, IL-1β and IL-10 were β = 0.137 ± 0.053, *P* = 0.020; β = 0.145 ± 0.054, *P* = 0.019; β = 0.182 ± 0.054, *P* = 0.0066; and β = 0.169 ± 0.055, *P* = 0.0090, respectively). The overall number of significant associations decreased from 7 (3 inverse, 4 positive) using model 1–6 (2 inverse, 4 positive) using model 2, representing a 14% decrease because of adjustment for VFMI.

**FIGURE 6:**
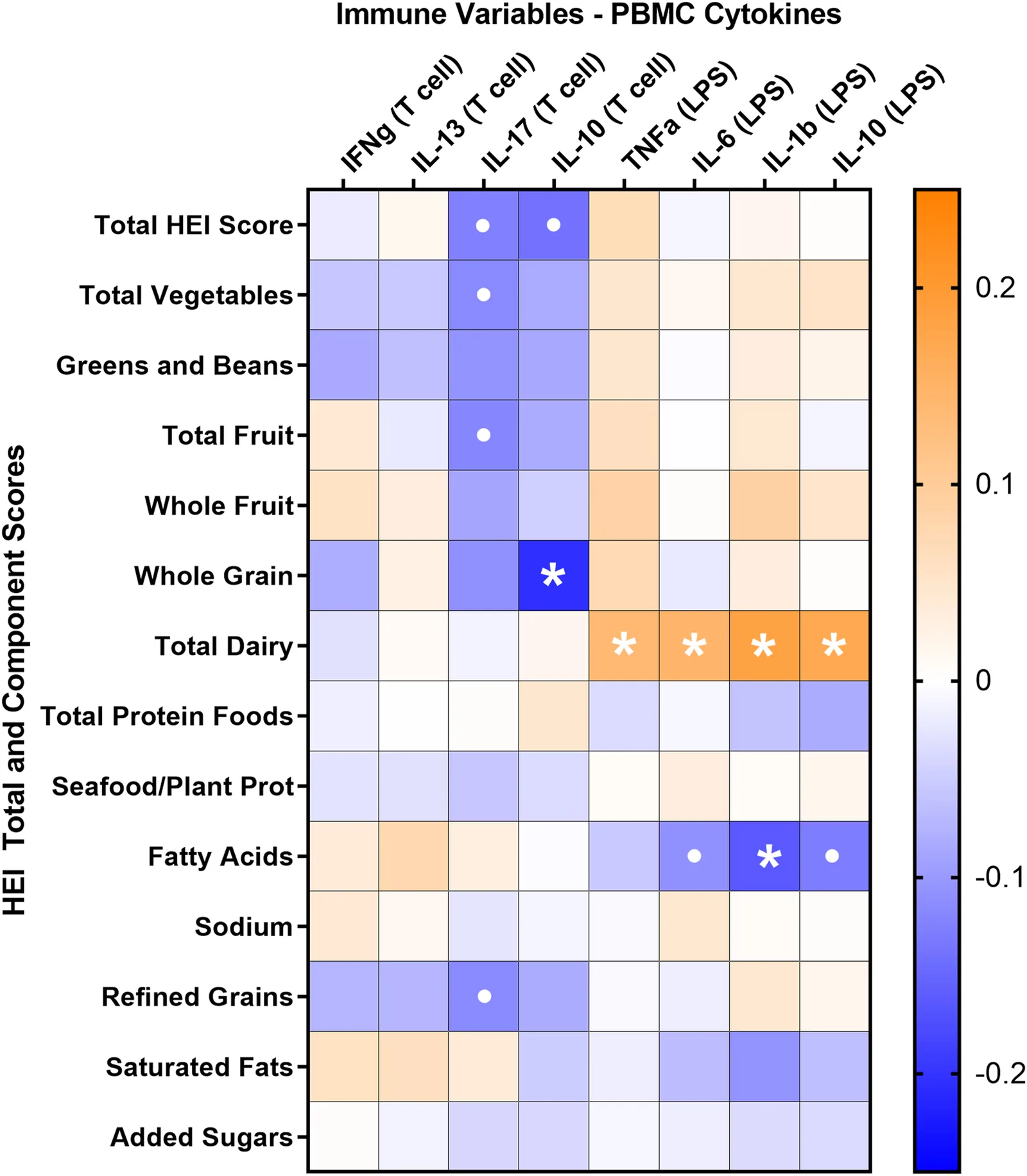
Heat map of standardized β-coefficients from general linear models showing association of total and component Healthy Eating Index (HEI) scores with 8 immune variables showing capacity of PBMC cultured ex vivo to produce cytokines following activation of T cells (IFN-g, IL-13, IL-17A, and IL-10) and activation with lipopolysaccharide (LPS) to produce TNF-a, IL-6, IL-1b and IL-10) adjusting for age, sex, visceral fat mass index, season (using month of year) and cytomegalovirus immune status. Significant associations with and without adjustment for multiple comparisons are shown with an asterisk (∗) or dot (^•^), respectively. Sample sizes for each analysis are shown in Supplemental Table 2, and β-coefficients, SEs, and P.values are shown in Supplemental Table 3.

## Discussion

### Plasma immune variables

In the present study, we examined the association of HEI-2015 and its 13 component scores with 19 plasma markers of inflammation. Several of these, including CRP, SAA, IL-6, TNF-α, ICAM-1, and VCAM-1 have also been examined in 7 previous studies using the HEI (12-18). In the present study, we found that the overall HEI-2015 score was not significantly associated with any of the 19 plasma markers using model 2, which included adjustment for VFMI. In model 1, without VFMI adjustment, the total HEI score and 3 component scores were all significantly inversely associated with plasma CRP. This dramatic difference between the 2 models results from the significant relationship between visceral adiposity and development of inflammation and elevation of plasma CRP, as recently reviewed [27]. Of the 7 previous studies that examined the association of HEI with plasma markers of inflammation [[12], [13], [14], [15], [16], [17], [18]], all included CRP as a dependent variable. Three of these 7 found statistically significant inverse associations between the original HEI [12,14] or HEI-2015 [18] and CRP. These 3 studies all adjusted for BMI as a proxy for adiposity and had the largest sample sizes (ranging from 1989–13,811), suggesting that diet can directly affect CRP concentration, but the effect size is such that large sample sizes are needed to find statistical significance. Other plasma markers were examined in >1 of these 7 studies (SAA, IL-6, TNF-α, ICAM-1, and VCAM-1), but the 2 largest studies [12,14] examined only CRP whereas the third study also examined IL-6 and TNF-α but these associations were not significant [18].

### Blood leukocytes

In the present study, as well as in the 1 other study using HEI-2015 [18], significant, inverse associations were seen between the overall HEI-2015 (as well as several component scores, including total vegetables, greens, and beans, whole grains, fatty acids, and saturated fats) with total leukocyte, neutrophil, lymphocyte, and monocyte concentrations. Including VFMI in the model in the present study and BMI in the study by Millar et al. [18] weakened these associations, though statistical significance remained for the inverse association of the overall HEI-2015 with total leukocytes and neutrophils in the study by Millar et al. [18] but not for any of the blood leukocyte variables in the present study. However, inverse associations were seen between the total vegetable and greens and beans component scores with lymphocytes in the present study with VFMI in the model. No association was seen between HEI-2015 and the neutrophil-to-lymphocyte ratio in the present study, but a study in Spanish older adults found an inverse association of the Spanish HEI with this ratio, although the analysis was not adjusted for BMI or other measures of obesity, which were found in the same study to be positively associated with the neutrophil-to-lymphocyte ratio [28].

### Innate leukocyte types and activation level

The principal findings with this data set were that the total vegetables component score was inversely associated with CD11b expression on all monocyte types as well as on neutrophils. To our knowledge, this is a novel observation that suggests that cellular activation resulting in CD11b expression is responsive to diet. CD11b is also known as a_M_ integrin and is part of the heterodimeric a_M_b_2_ integrin (CD11b/CD18), which is found on monocytes, macrophages, granulocytes, and NK cells [29]. CD11b itself is responsible for binding to ICAM-1 on other immune cells as well as to fibrinogen and complement components, whereas CD18 plays a role in cell migration. Thus, a_M_b_2_ integrin is important for leukocyte adhesion and migration and plays a key role in inflammation. Increased expression of CD11b on such cells has been linked to risk of cardiovascular disease [22] and complications of diabetes [24,25]. Higher intake of total vegetables may dampen activation of monocytes and neutrophils as an intermediate factor in improving health.

### T-cell percentages, subtypes, and activation levels

Neither model 1 nor model 2 revealed significant associations of overall HEI or component scores with these T-cell variables, suggesting that the T-cell component of the adaptive immune system is not modulated by these dietary factors. Although it is well known that malnutrition negatively affects thymic function, T-cell concentrations, and responses [30], variations in nutritional quality, as captured by the HEI, appear not to affect these components of adaptive immunity.

### T-helper cells, B cells, and NK cells

A higher refined grains component score (indicating lower intake of refined grains and possibly higher intake of fiber-containing whole grain) was inversely associated with NK T-cell percent in model 2. NK T-cells increase with high-fat diets in mouse studies and are associated with cardiovascular disease in mouse models and human studies [31]. One mouse study has shown that dietary fiber decreases invariant NK T-cell function via production of short-chain fatty acids [32], suggesting a possible mechanistic link between a higher refined grains score and lower NK T-cell percent.

### Production of cytokines by PBMC cultures

A higher whole grain component score was inversely associated with production of the regulatory cytokine IL-10 by T cells in both model 1 and 2, but no associations were seen for the other 3 T-cell cytokines, IFN-g, IL-13, and IL-17. Lower T-cell production of IL-10 is consistent with lower Treg development, which is counterintuitive given the known association of higher fiber intake (which is found in whole grain) with increased Treg cell development at mucosal sites [33,34], although IL-10 can be produced by multiple types of T cells. Further work is needed to determine if there is a plausible mechanistic association between whole grain intake and lower IL-10 production by T cells.

Perhaps the most striking observation in this variable set is the positive association of the total dairy component score with production of all 4 cytokines measured following stimulation with bacterial LPS (TNF-α, IL-6, IL-1β, and IL-10). Because these variables correlate strongly with one another (data not shown), it is reasonable to think of this observation as an association with responsiveness of PBMC to LPS stimulation, which occurs via the TLR4 pathway [35], rather than an association with specific cytokines that may be viewed as either pro- (TNF-α, IL-6, and IL-1β) or anti-inflammatory (IL-10). These results suggest that intake of dairy food might promote health by enhancing the response to bacterial challenges, as represented in the present study by LPS stimulation. The fatty acids score (indicating higher intake of n–3 [ω-3] PUFA or MUFA) also showed an inverse association with production of one LPS-stimulated cytokine, IL-1b. Thinking mechanistically, it is interesting to note that saturated fatty acids, which are found in some dairy foods [36], may directly trigger cell activation via TLR-4, and at least one long-chain n–3 PUFA, DHA, which is not typically found in dairy foods, can inhibit activation via TLR4 [37]. Although these countervailing mechanisms might help explain these observations, no association between the saturated fat component score was seen with LPS-stimulated cytokine production. Further studies are needed to explore a mechanism for these observations, as our observational study design cannot be used to infer causality. In general, it should be noted that dairy foods do not increase markers of chronic inflammation, and whereas anti-inflammatory activity has been noted in some studies, current research does not suggest an overall “anti-inflammatory” profile according to recent reviews [36,38,39].

### Strengths and Limitations

Strengths of the study include the focus on “healthy” adults with exclusions for diagnosed chronic disease, although the wide BMI range did include, by design, many individuals with obesity, which is itself a health risk. The sample size was relatively large (*n* = 362 for the present analysis) for a study with such extensive dietary and physiologic characterization (e.g., assessment of VFMI) and used a novel set of markers covering many aspects of immune function [21]. Diet was characterized by 24 h recalls rather than a less quantitative method such as a food frequency questionnaire. The statistical analysis adjusted for many covariates that affect immune markers (including VFMI, age, sex, activity, seasonality, and CMV infection status) to identify the association of HEI-2015 more directly with immune function. Limitations of the study compared with others assessing HEI and inflammation are that the sample size is smaller than other studies (∼2000 or more participants), which did identify significant associations of HEI with some standard markers of inflammation and immune activation [12,14,18] where no associations were seen here. It is possible, however, that the present cohort, selected because they were “healthy,” differs significantly from those of other studies that had less selective screening criteria. It should also be noted that the effect sizes seen here are relatively small. The absolute value of the statistically significant β-coefficients (analogous to correlation coefficients), which are useful to estimate effect sizes [40], range from 0.122 to 0.204, indicating explanation of ∼2% to 4% of the variation in the dependent immune variable.

In conclusion, our finding that the overall HEI-2015 score was not significantly associated with any of these 88 immune variables after adjustment for VFMI is consistent with data in the literature showing that visceral adiposity itself is a mediator of the impact of poor dietary patterns on inflammation. However, our finding that the HEI component scores for whole grains, refined grains, total vegetables, fatty acids, and total dairy were significantly associated with higher or lower levels of immune activation in a model with VFMI adjustment suggests that focusing on specific components of the overall dietary pattern may be a beneficial approach to develop dietary guidance.

## Author contributions

The authors’ responsibilities were as follows – CS was involved in project conception, development of overall research plan, study oversight, wrote the paper, and had primary responsibility for final content. XJ was involved in development of immunologic assays, conducted research, and cleaned data for database entry. BG conducted research, including collection and analysis of DXA data. JP performed statistical analysis. All authors have read and approved the manuscript.

## Data Availability

Requests for data from the Nutritional Phenotyping Study used in this analysis should be made via email to the corresponding author. Requests will be reviewed quarterly by a committee consisting of the study investigators.

## Funding

This work was supported with funding from a USDA Agricultural Research Service (Project numbers 2032-51530-026-000-D, 2032-51530-025-00-D, 2032–53000–001-00-D and 2032-51530-022-00-D) as well as the National Center for Advancing Translational Sciences (NCATS), and National Institutes of Health (NIH) through a grant, UL1 TR000002.

## Conflict of interest

Charles Stephensen is on the Editorial Board for The Journal of Nutrition and played no role in the Journal's evaluation of the manuscript. All other authors report no conflicts of interest.
