# Supplementary material for: Association of Healthy Eating Index-2015 Total and Component Scores with Measures of Inflammation and Immune Activation in Healthy Adults
Source: J Nutr. 2025 Jan 7;155(3):994–1004. doi: 10.1016/j.tjnut.2025.01.005 (PMC13639635; doi:10.1016/j.tjnut.2025.01.005)
Supplement: Supplementary file 1 [file mmc2.docx]

**Supplemental Figure 1.** Body mass index (BMI) and visceral fat mass index (VFMI) mean and standard deviation values for all study participants with available data (All), males (M) and females (F), the three age recruitment categories (A1, 18-33; A2, 34-49; and A3 50-65 y), and the three BMI recruitment categories (B1, 18.5-25; B2, 25-30; and B3, 30-45 kg/m^2^). Significant differences between groups are indicated with letter superscripts where means with different letters differ from one another. Sample sizes for BMI data are: All, 393; M/F, 184/209; A1/A2/A3, 140/130/123; and B1/B2/B3, 145/144/104. Sample sizes for VFMI data are: All, 390; M/F, 181/209; A1/A2/A3, 138/130/122; and B1/B2/B3, 145/142/103.

**Supplemental Figure 2.** Activity levels (mean and standard error, minutes/day) in four categories (sedentary, light, moderate and vigorous) for all study participants with available data (All), males (M) and females (F), the three age recruitment categories (A1, 18-33; A2, 34-49; and A3 50-65 y), and the sex-specific visceral fat mass index (VFMI) tertiles (F1, F2 and F3, lowest through highest) described in Methods. Significant differences between groups are indicated with letter superscripts where means with different letters differ from one another. Sample sizes for each category are: All, 372; M/F, 176/196; A1/A2/A3, 129/126/117; and F1/F2/F3, 127/124/121.

**Supplemental Figure 3.** Total Healthy eating Index (HEI) score and component scores (mean and standard deviation, minutes/day) for all study participants with available data (All), males (M) and females (F), the three age recruitment categories (A1, 18-33; A2, 34-49; and A3 50-65 y), and the sex-specific visceral fat mass index (VFMI) tertiles (F1, F2 and F3, lowest through highest) described in Methods. Significant differences between groups are indicated with letter superscripts where means with different letters differ from one another. Sample sizes for each category are: All, 378; M/F, 172/206; A1/A2/A3, 133/126/119; and F1/F2/F3, 125/127/126.

**Supplemental Figure 4.** Heat map of standardized beta coefficients from general linear model 1 showing association of total and component Healthy Eating Index (HEI) scores with 19 plasma immune variables adjusting for age, sex, season (using month of year) and cytomegalovirus immune status. Significant associations with and without adjustment for multiple comparisons are shown with an asterisk (*) or dot (^●^), respectively. Sample sizes for each analysis are shown in Supplemental Table 2 and beta-coefficients, standard errors and p-values are shown in Supplemental Table 3.

**Supplemental Figure 5**. Heat map of standardized beta coefficients from general linear model 1 showing association of total and component Healthy Eating Index (HEI) scores with 15 complete blood count immune variables adjusting for age, sex, season (using month of year) and cytomegalovirus immune status. Significant associations with and without adjustment for multiple comparisons are shown with an asterisk (*) or dot (^●^), respectively. Sample sizes for each analysis are shown in Supplemental Table 2 and beta-coefficients, standard errors and p-values are shown in Supplemental Table 3.

**Supplemental Figure 6.** Heat map of standardized beta coefficients from general linear model 1 showing association of total and component Healthy Eating Index (HEI) scores with 11 immune variables showing activation levels of monocyte types (Alternative, Intermediate and Classical), neutrophils and eosinophils adjusting for age, sex, season (using month of year) and cytomegalovirus immune status. Significant associations with and without adjustment for multiple comparisons are shown with an asterisk (*) or dot (^●^), respectively. Sample sizes for each analysis are shown in Supplemental Table 2 and beta-coefficients, standard errors and p-values are shown in Supplemental Table 3.

**Supplemental Figure 7.** Heat map of standardized beta coefficients from general linear model 1 showing association of total and component Healthy Eating Index (HEI) scores with 24 immune variables for CD4 and CD8 T-cell types (memory, central memory, effector memory) and subsets showing activation (HLA-DR and CD38) and exhaustion (PD1) marker expression adjusting for age, sex, season (using month of year) and cytomegalovirus immune status. Significant associations with and without adjustment for multiple comparisons are shown with an asterisk (*) or dot (^●^), respectively. Sample sizes for each analysis are shown in Supplemental Table 2 and beta-coefficients, standard errors and p-values are shown in Supplemental Table 3.

**Supplemental Figure 8.** Heat map of standardized beta coefficients from general linear model 1 showing association of total and component Healthy Eating Index (HEI) scores with 11 immune variables for CD4 T-cells (TH1, Th2, TH17), NK cells and B cells adjusting for age, sex, season (using month of year) and cytomegalovirus immune status. Significant associations with and without adjustment for multiple comparisons are shown with an asterisk (*) or dot (^●^), respectively. Sample sizes for each analysis are shown in Supplemental Table 2 and beta-coefficients, standard errors and p-values are shown in Supplemental Table 3.

**Supplemental Figure 9.** Heat map of standardized beta coefficients from general linear model 1 showing association of total and component Healthy Eating Index (HEI) scores with 8 immune variables showing capacity of PBMC cultured ex vivo to produce cytokines following activation of T cells (IFN-γ, IL-13, IL-17A and IL-10) and activation with lipopolysaccharide (LPS) to produce TNF-a, IL-6, IL-1β and IL-10) adjusting for age, sex, season (using month of year) and cytomegalovirus immune status. Significant associations with and without adjustment for multiple comparisons are shown with an asterisk (*) or dot (^●^), respectively. Sample sizes for each analysis are shown in Supplemental Table 2 and beta-coefficients, standard errors and p-values are shown in Supplemental Table 3.
